# Examining the structure validity of the Pittsburgh Sleep Quality Index among female workers during New Zealand’s initial COVID-19 lockdown

**DOI:** 10.1007/s41105-023-00509-6

**Published:** 2024-01-19

**Authors:** Margrethe Helles, Richard Fletcher, Mirjam Münch, Rosemary Gibson

**Affiliations:** 1 School of Psychology, Massey University, Palmerston North, New Zealand; 2 Centre for Chronobiology, Psychiatric Hospital of the University of Basel, Basel, Switzerland; 3 Research Cluster Molecular and Cognitive Neurosciences, Department of Biomedicine, Basel, Switzerland; 4 Sleep/Wake Research Centre, School of Health, Massey University, Wellington, New Zealand; 5 Research Centre for Hauora and Health, College of Health, Massey University, Wellington, New Zealand

**Keywords:** Confirmatory factor analysis, Employed woman, Pandemic, PSQI; sleep measure

## Abstract

Sleep is important for good physical and mental health. The COVID-19 pandemic lockdown created a unique context that impacted psychological and social drivers for sleeping well. The Pittsburgh Sleep Quality Index (PSQI) is a widely used measurement tool assessing subjective sleep quality. The traditional model of the PSQI (a one-factor model), whilst validated and used across different populations, has also been questioned with regards to data fit and representativeness of its global score in different social and work-related circumstances. Examination of the structure validity of the PSQI in the unique context of the pandemic has been scarce. This study determined the PSQI structure validity amongst employed women considered to experience increased stressors during the pandemic lockdown. The subjectively reported PSQI data from 498 female workers (mean age 44.6 years) collected during New Zealand’s first national COVID-19 lockdown (April, 2020) was used. Confirmatory factor analyses compared the original one-factor model of the PSQI with the two- and three-factor models used by Jia et al. (2019) within this pandemic context. Results showed that the two-factor model provided a superior fit of the PSQI data compared to the original one-factor or a three-factor model. These findings suggest that a sub-score of the PSQI with two factors appears to be better at describing the sleep quality of healthy working women during the constrained situation of the pandemic lockdown compared to a single global sleep quality score. This indicates the importance of considering the validity of subjective sleep measures when used within unique social contexts and stressors.

## Introduction

Sleep is an important foundation for good physical and mental health as it supports every system in the body including metabolic, immune, cognitive function and emotional regulation [1]. Sleep quality is a complex construct and includes quantitative characteristics such as sleep duration, number and length of awakenings, sleep regularity, as well as subjective aspects like perceived sleep depth and daytime functioning. Sleep health is multifaceted and influenced by biology behaviours as well as contextual and social factors including age, gender, social engagement, socio-economic and work status [1].

Within New Zealand (NZ) the COVID-19 pandemic and the mandatory lockdown that followed abruptly changed daily life for everyone, nationally as it was the case globally. In many countries, public health authorities enforced social restrictions such as closures of non-essential workplaces, school and day-cares as an epidemiological containment strategy impacting daily routines, physical activity, and other drivers of sleep. In pursuit of an elimination strategy, the early lockdowns in NZ were considered some of the strictest in the world whilst the infection rates remained low [2]. During the strictest level of lockdown (Level 4), only essential services were open, and the population (except essential workers) had to remain within their ‘house bubbles’ (close family members only), for 33 days. In the less rigorous lockdown level (Level 3) gatherings were restricted to up to 10 people [3]. Research suggests the stress associated with the COVID-19 lockdowns and associated social restrictions significantly affected sleep quality on a global scale [4–6]. A meta-analysis found the prevalence of sleep disturbances during the pandemic was higher in females (41%) compared to males (31%) [7]. Furthermore, females were identified as more likely to have taken on the task of home-schooling and full-time care for children whilst fulfilling work obligations from home [8]. Therefore, research focusing on the impact of the pandemic on sleep and wellbeing within stratified samples of working females are warranted.

A measure for sleep quality used extensively in sleep research is the Pittsburgh Sleep Quality Index (PSQI) [9] which is the focus of this study. The PSQI is a self-report questionnaire comprised of 19 items that assess subjective sleep quality during the previous month. It is one of the most widely used self-report measures for assessing subjective sleep quality and is well validated in clinical and community populations. Typically, all variables contribute to calculating one or more of the seven component scores which indicate various indicators of sleep health (or disturbance). They are then collapsed again to form a global score (between 0 and 21) making it an easy applicable instrument for research and clinical practice [10]. However, more recently, the performance of self-reported sleep scales has been debated. For example across time, clinical profiles, and age-groups or different cultures [11]. This calls to question how sleep quality scales such as the PSQI performed during the pandemic lockdown (a context which dramatically changed routines and behaviours). Few studies have assessed the PSQI structure validity during the COVID-19 pandemic [12] and in special populations such as working females.

To date, most researchers use the PSQI [9] with the global score (i.e. as a one-factor model) aiming to capture all attributes of subjective sleep quality. Prior the COVID-19 pandemic, some studies have shown that multifactorial models for PSQI can improve the probability of detailing the severity of sleep disturbance because components are represented and weighted across separate domains [13, 14]. Fabbri et al.’s recent systematic review of the PSQI psychometric properties reported good internal reliability and validity however, different factorial structures were noted; six papers reported a single dimension, six studies indicated a two-factor model and two papers a three-factor model [11]. Manzar et al. (2018) [15] conducted another meta-review of the PSQI factor structure, summarising 30 distinct PSQI models proposed in the literature. However, due to methodological discrepancies between the 45 studies included (for example, adequacy of sample evaluation, application of factor analysis, variation in software used, tests conducted, and outputs reported), the application of these findings is limited. To overcome these shortcomings, the authors proposed methodological guidelines for examining the structure validity of the PSQI in future studies. This is especially the case for habitual sleep quality under different environmental and social constraints. The internal factor validity may differ between circumstances that vary compared to those within which it was initally validated. Based on Manzar et al.’s (2018) [15] suggestions, Jia and colleagues [14] re-examined the PSQI structural validity in a large (*N* = 2189) non-clinical sample of Americans (64% female, mean age 35.9 years, SD = 12.2) by testing one, two, and three-factor models. Their results indicated that the two-factor model (which they named ‘sleep efficiency’ and ‘sleep latency’) and three-factor models (which they named ‘sleep efficiency’, ‘sleep latency’ and ‘sleep quality’) were statistically superior to the one-factor PSQI (i.e. the original global score). Because Jia et al.’s [14] models used a rigorous methodology to improve discrepancies in the validation literature, especially when the PSQI is used under different environmental and social constraints as described above, a similar approach was used in this study. Note, due to the naming of factors by Jia et al., the terminology around ‘sleep latency’ and ‘habitual sleep efficiency’ are used differently here compared to clinical definitions used elsewhere [16].

These works indicate the importance of considering the internal reliability of the PSQI items in various research contexts. The COVID-19 lockdowns created a situation where, due to the social restrictions, self-reported sleep status changed [4–6]. However, the interpretation and reliability of responses to items within surveys such as the PSQI may also have been affected. For example, the reliability of estimating bed and sleep times may have been hindered because, for many, the external drivers to physically attend work or educational facilities were dropped (and therefore regularity and remembrance of routines and use of aids such as alarm clocks reduced). Furthermore, it is anticipated that how participants interpret and estimate frequencies of issues such as having “trouble staying awake while driving, eating meals, or engaging in social activity?” may also be questionable during a period when confined to their homes with limited social engagement, despite having low infection rates (as was the case in NZ).

Given prior studies questioning the use and interpretation of the PSQI in various conditions [11, 14, 15], the current study aimed to assess the factorial validity of the PSQI amongst a unique population of working females during New Zealand’s first national lockdown (April–May 2020). This is novel as few studies have examined the structural validity of PSQI during the context of the COVID-19 pandemic restrictions. It is also the first study to assess factorial validity in this population who, as outlined above, have unique factors affecting their sleep. The structure validity of the original one-factor model (hypothesised to be superior due to its common-use, validity, and reliability across different populations and contexts prior to the pandemic) was evaluated against Jia et al.’s (2019) [14] two- and three- factor models to evaluate which has the best model data fit within this unique context and population.

## Methods

### Participants and data collection

The data derived from an existing dataset (for reports from the entire dataset please see: [17]). The original online survey (*‘Sleep and Well-being in NZ during COVID-19 Pandemic Restrictions Survey’*) was launched through Qualtrics from the 11th of April to 11th of May 2020, covering New Zealand’s most rigorous lockdown restrictions (Level 4 and 3). The survey was advertised via social media, press-releases, national television, and radio. Of the original sample (*N* = 723), 69% (*N* = 498) self-identified as female workers aged over 18 years (age range: 21–83 years) and were included in the present analyses. Workers were defined as participants who reported working full-time, part-time, or as self-employed/contractors prior to lockdown.

### Measures

Demographic variables included age, education, marital, employment, lifestyle, and health status (described in full in [17]). Subjective sleep quality was assessed using the PSQI, a self-report questionnaire comprised of 19 items concerning sleep during the previous month. These items are used to compute seven component scores: (1) Sleep quality; (2) sleep latency; (3) sleep duration; (4) habitual sleep efficiency; (5) sleep disturbances; (6) use of sleep medication; and (7) daytime dysfunction (see Buysse,1989 [9] for specific compositions of each score). Each component was weighted equally on a 0 to 3 scale and summed to provide a one-factor global PSQI score ranging from 0 (no sleep problems) to 21 (severe sleep problems) with scores > 5 considered indicative of 'problem sleep' according to the original validation and scoring reference [9]. In a pre-pandemic context, the PSQI had good internal reliability (*α* = 0.83) [9].

### Statistical analysis

Confirmatory factor analysis (CFA) was used to examine the PSQI factor models. Then the original one-factor model (named ‘sleep quality’ including all seven PSQI component scores) was compared with sub-scored multifactorial models as developed and labelled by Jia et al. (2019) using exploratory factor analysis [14]. Jia et al. applied a re-grouping of the original seven PSQI components and binned them differently. Firstly, a two-factor model was developed. To ensure accurate replication, the specifics of the two-model structure was reported by the original authors by personal communication (March 4th, 2022) and involves one factor being named ‘sleep efficiency’ (comprised of original PSQI component scores for: sleep duration and habitual sleep efficiency), and the second factor being named ‘sleep latency’ (comprised of original PSQI component scores for: sleep quality, sleep latency, sleep disturbances, use of sleep medication, and daytime dysfunction). Then a three-factor model was developed with one factor being named ‘sleep efficiency’ (comprised of original PSQI component scores for: sleep duration and habitual sleep efficiency), another being named ‘sleep latency’ (comprised of original PSQI component scores: sleep latency and use of sleep medication), and the third factor being named ‘sleep quality’ (comprised of original PSQI component scores: sleep quality, sleep disturbances, and daytime dysfunction).

The estimation method used by Jia et al. as well as for the present study, was the Maximum Likelihood method which assumes multivariate normality of the observed variables and requires large sample sizes [18]. Skewness and kurtosis of the PSQI variables was determined to assess the normality of PSQI with values. For the analysis, all PSQI variables had to be smaller or equal to 3.0 since values greater than 3.0 indicate severe skewness and 8.0–20.0 as severe kurtosis [19]. Due to the study design, all PSQI items were mandatory within the survey. Therefore, there was no missing data. Full information maximum likelihood estimation, within Analysis of Moment Structures (AMOS), was employed using all available data to estimate the model parameters. Results using this method have shown to produce unbiased parameter estimates and are generally the default option in many software programs.

The fit indices to determine the adequacy of the hypothesised data-model fit included the Comparative Fit Index (CFI) and Tucker-Lewis Index (TLI), with values close to 0.95 or greater represent a well-fitting model [20]; Root-Mean-Square Error of Approximation (RMSEA) which are less than 0.06 indicate a good fit [20], 0.08 a mediocre fit and greater than 0.10 a poor fit [21]. The precision of RMSEA estimates was reported with 0.90% confidence intervals. No post hoc modifications were made to the models. All descriptive statistics were analysed using IBM SPSS 29.0. The CFA was conducted with IBM Amos version 29.0. The internal-consistency reliability was assessed by Cronbach’s alpha coefficient.

## Results

### Demographic characteristics

The participants included were 498 female workers living across NZ during the first 2020 COVID-19 lockdown. A description of the participants, demographic information and the PSQI items can be found in Table 1. The skewness of the global PSQI scores was 0.87 and kurtosis 0.66 indicating a normal distribution (mean = 6.6, Standard Deviation; SD = 3.5) Scores indicative of ‘poor sleep’ (PSQI > 5) were present amongst 54.2% of the participants.

**Table 1:** Descriptive details of participants and PSQI items (*N* = 498)

	*n*	%	Mean	(SD)	Median	(IQR)	Skewness	Kurtosis
Age (years)	498		44.56	(12.62)	44.50	(19.00)	0.21	− 0.65
Married / de facto relationship	344	69.10						
New Zealand European ethnicity	326	72.80						
Tertiary level education / qualification	395	79.30						
Employment status								
Full time work	316	63.50						
Part time work	122	24.50						
Self-employed / contractor	45	9.00						
Multiple work roles	15	3.00						
Health status								
Excellent	131	26.30						
Very good	217	43.60						
Good	108	21.70						
Fair–poor	42	8.40						
PSQI								
Sleep duration (hrs)^a,b^			7.69	(1.30)	7.92	(1.50)	− 1.06	2.71
Bedtime (hh:mm)^b^			22:31	(1:12)	22:30	(1:16)	0.31	1.8
Risetime (hh:mm)^b^			7:55	(1:12)	7:48	(1:16)	0.74	2.55
Sleep efficiency (%)^b^			82.55	(13.04)	86.05	(13.39)	− 1.82	4.42
Sleep latency (mm)^c^			30.40	(29.38)	20.00	(27.86)	1.64	2.07
Cannot get to sleep within 30 min?^c^								
Not during the past month	151	30.30						
Less than once a week	108	21.70						
Once or twice a week	95	19.10						
Three or more times a week	130	26.10						
Use sleeping medications?^d^								
Not during the past month	430	86.3						
Less than once a week	19	3.8						
Once or twice a week	33	6.6						
Three or more times a week	35	7.0						
Trouble staying awake while driving, eating meals, or engaging in social activity?^5^								
Not during the past month	393	78.90						
Less than once a week	50	10.00						
Once or twice a week	32	6.40						
Three or more times a week	9	1.80						
…Enthusiasm to get things done?^e^								
No problem at all	60	12.00						
Only a very slight problem	179	35.90						
Somewhat of a problem	179	35.90						
A very big problem	66	13.30						
How would you rate your sleep quality overall?^f^								
Very good	87	17.50						
Fairly good	227	45.60						
Fairly bad	152	30.50						
Very bad	32	6.40						
Sleep disturbance (sum 0–27)^g^			8.74	(4.73)	8.00	(7.00)	0.45	− 0.21
PSQI global score (0–21)	498		6.59	(3.53)	6.00	(5.00)	0.87	0.66
PSQI ‘poor sleeper’ (PSQI > 5)	270	54.20						

*n* sample size, *SD* standard deviation, *IQR* interquartile range, *Hrs* hours, *hh:mm* hours and mintues, *mm* minutes, *PSQI* pittsburgh sleep qulaity Index

^a^Contributes to component score: “sleep duration”

^b^Contributes to component score “habitual sleep efficiency”

^c^Contributes to component score “sleep latency”

^d^Contributes to component score “Use of sleep medications”

^e^Contributes to component score “daytime dysfunction”

^f^Contributes to component score”sleep quality”

^g^Contributes to component score “sleep disturbances”

### Internal-consistency reliability

Reliability estimates for the PSQI one-factor sleep quality model was 0.73 (Cronbach’s α). For the two-factor model the α estimates were 0.73 for the sleep duration factor and 0.64 for the sleep latency factor. For the three-factor model the α estimates were 0.73 for the sleep efficiency factor, 0.38 for the sleep latency factor, and 0.63 for the sleep quality factor.

### Confirmatory factor analysis

The CFA model fit indices are displayed in Table 2. Of the three models specified, the two-factor model was deemed to be the best fitting. This was because, across the three fit indices, the estimates were better compared to the other models. The model fit indices for the two-factor model showed the RMSEA of 0.08 (90% C.I.: 0.06, 0.11) and the CFI and TLI of 0.94 and 0.90 indicated a good fit.

**Table 2:** Model Fit Indices from CFA results

	SB(χ2)	*Df*	RMSEA (90% C.I.)	CFI	TLI
PSQI One-factor model	191.47***	14	0.16 (0.14, 0.18)	0.75	0.63
PSQI Two-factor model	56.86***	13	0.08 (0.06, 0.11)	0.94	0.90
PSQI Three-factor model	80.55***	12	0.11 (0.09, 0.13)	0.90	0.83

*PSQI Global* pittsburgh sleep quality index, *SB(χ2)* Chi-square, *Df* degrees of freedom, *RMSEA* root-Mean-square error of approximation, *CFI* comparative fit index, *TLI* tucker-lewis index

*p < 0.05, **p < 0.01, ***p < 0.001

**Fig. 1:**
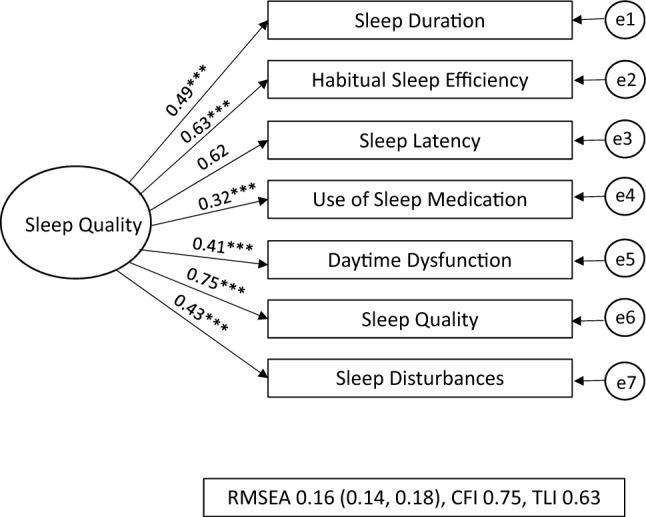
PSQI One-Factor Model for NZ female workers during lockdown, One-Factor model where all seven PSQI components are hypothesised as one-factor model being ‘sleep quality’ [9]. RMSEA: Root-Mean-Square Error of Approximation 0.16, CFI: Comparative Fit Index 0.75, and the TLI: Tucker-Lewis Index 0.65 all suggested poor fit, e1-e7 = error. *p < 0.05, **p < 0.01, ***p < 0.001

**Fig. 2:**
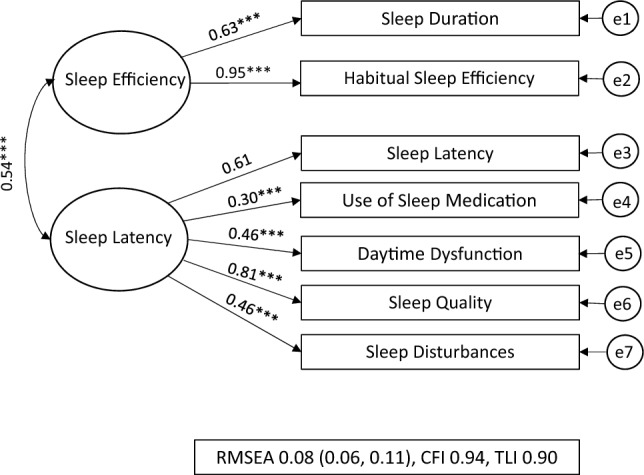
PSQI 2-Factor Model for NZ female workers during lockdown Two-factor model was specified using the seven PSQI components into two separate correlated factors being ‘sleep efficiency’ or ‘sleep latency’ as per Jia et al. [14]

**Fig. 3:**
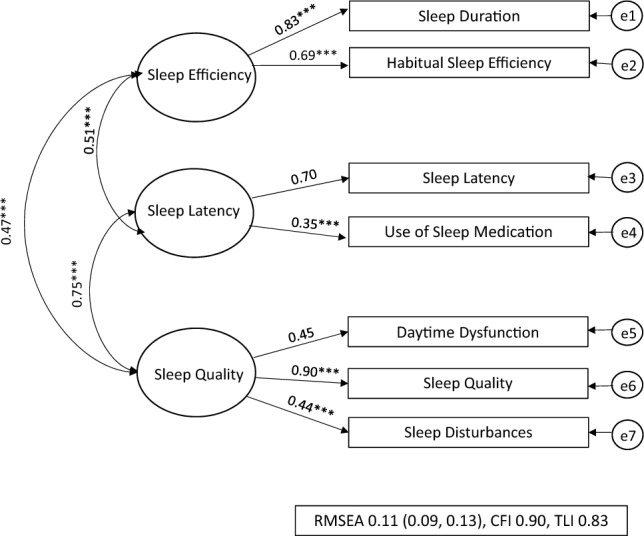
*PSQI 3-Factor Model for NZ Female Workers* The three-factor model was specified using the seven PSQI components into three separate correlated factors ‘sleep efficiency’, ‘sleep latency’, or ‘sleep quality’ as per Jia et al. [14]. RMSEA: Root-Mean-Square Error of Approximation, CFI: Comparative Fit Index, TLI: Tucker-Lewis Index all suggested lower (poor-adequate) fit compared to the two-factor model, e1-e7 = error. *p < 0.05, **p < 0.01, ***p < 0.001

The three different models are shown in Figs. 1, 2, 3 presented with their standardised factor loading and correlations. The standardised factor loadings for the two factors (sleep efficiency and sleep latency) and the PSQI item scores ranged from 0.30 (use of sleep medication) and 0.95 (habitual sleep efficiency). The latent factor correlation between the sleep quality and sleep factors was 0.54.

RMSEA: Root-Mean-Square Error of Approximation, CFI: Comparative Fit Index, TLI: Tucker-Lewis Index all suggested good model data fit, e1-e7 = error. *p < 0.05, **p < 0.01, ***p < 0.001.

## Discussion

This study examined the structure validity of the PSQI in a sample of working females during the first national COVID-19 lockdown in NZ. The PSQI is one of the most widely used assessment tools for measuring subjective sleep quality. However, evidence for its structure validity during the COVID-19 pandemic is needed to help facilitate and advance reliable understandings of sleep, and sleep quality amongst a range of behavioural, social, and health factors during this unique probably stressful context.

Previous research has validated the original PSQI one-factor model [22]. However, it was validated within American college students as participants who were predominantly young, white, of high socioeconomic status, and included both sexes. Furthermore, limitations of the PSQI one-factor model (in relation to its structure validity) have been noted for use in the general population prior to the pandemic [11, 15]. Other evidence suggests that subjective sleep quality may be better assessed by the PSQI two-factor model [23–27]. The present findings corroborate this, at least under the general constraint of a pandemic situation. For this sample of working females within the context of New Zealand’s pandemic lockdown, the traditional one-factor model did not fit as well as the two-factor model. The unique context of the pandemic lockdowns with their strict social restrictions (particularly in countries like NZ) created a situation within which the routines of waking and sleeping life were affected [2]. Such changes have been identified as impacting sleep [4–6, 17] and potentially how participants interpret questions concerning their wellbeing [28]. The female worker population may also be highly unusual compared to characteristics of other populations that have previously been used to validate the PSQI. For example, the added stress many females were facing during the lockdown by having a higher proportion of the childcare and home-schooling burden whilst also working, may have led to compromised sleep patterns [5, 29, 30].

Results from this study suggest that multifactorial models of the PSQI might provide stronger data fit of the seven components compared to the original one-factor model. This is in line with Jia et al.’s (2019) findings [14]. However, out of the three hypothesised models, only the two-factor model met the threshold for good model fit to the PSQI data of the present NZ female worker population. The two-factor model had a good internal consistency for the latent factor ‘sleep efficiency’ and but below threshold reliability for latent factor ‘sleep latency’ (for recommended thresholds see [31]). These results likely reflect the nature and weighting of the scoring system of the seven individual components of the PSQI in the social constraints of the pandemic. As the base scoring system uses one or more subjective sleep variables to inform each of the seven sleep-related component scores [9], it is difficult to truly determine the contribution of one specific variable within measures like the PSQI when it has been combined and collapsed into component scores and then a single global score (or in the present case binned into two or three-factor sub scores) [32]. This may be problematic when applied in contexts like the pandemic, where the variables of self-reported sleep of the global PSQI have been affected differently than under ‘normal’ situations. In addition, some individuals reported better sleep quality compared to pre-pandemic estimates, while for others it was worse [17]. Similarly, for some, sleep disturbances associated with their home and sleeping environment or work schedules improved during lockdown, whereas for others it became more challenging [3–6, 17].

In this pandemic context, the PSQI with revised factors differentiating between ‘sleep efficiency’ (incorporating PSQI component scores for sleep duration and habitual sleep efficiency) and ‘sleep quality’ (incorporating PSQI component scores for sleep latency, use of medications, daytime dysfunction, sleep disturbances, and self-rated sleep quality) provided a better fit than the traditional single factor ‘sleep quality’ score (incorporating all seven PSQI component scores). This two-factor model may have been a superior fit for this population at this time due to its ability to account for the nuanced and sometimes contradictory differences observed in reports of changes to times in bed and sleep durations during the pandemic compared to perceptions of sleep status and changed factors disturbing sleep [3–6, 17]. In other words, for this sample of working women, the specific stress situation of the pandemic did not appear to affect all components of the PSQI in the same way as would be expected under ‘normal’ conditions. These findings indicate the importance of understanding the factor structure within component-based scales of the PSQI (and other such measures using a similar approach to component scoring) prior to its application and interpretation in general research settings. Greater attention towards the analysis and interpretation of the PSQI is required to better interpret and differentiate the contribution of single variables in both the estimation of reliability and the factor models.

There are several considerations concerning the present study which may inform future research. First, the original online survey was collected within a limited timeframe (≈30 days) and aimed at recruiting a heterogenous sample. However, the sample was a convenience sample with majority being female, highly educated, of NZ European ethnicity and therefore not representative of the NZ population limiting generalisability [17]. This study worked to the strength of the dataset and focused on a stratified sample of working females. The pandemic was unprecedented with a limited time prior the announcement of the lockdown, yet the original survey managed to collect the data at this time as opposed to other research which used retrospective surveys (e.g. [33]). Second, there was an underrepresentation of Māori and Pasifika in the cohort compared to the NZ population indicating response bias. Previous research has identified disparity in these populations with lower socio-economic status, sleep disturbances and mental health problems [34]. Thus, the prevalence of sleep problems is likely greater in the NZ population than reported here. Finally, as this is the first PSQI-related structural validity study in NZ, it is not possible to compare the difference in validity of the scale among working NZ females to pre-pandemic. Therefore, it is recommended that future research is required to validate the PSQI in a NZ representative sample and explore the factor structure as well as cut-off scores to identify problem sleep (in the various factor structures) for unique populations and contexts across genders, different age groups, and ethnicities separately.

Consideration of which subjective variables drive sleep-related concept outcomes is important. Factors of the scored components are typically labelled to represent the composition of its items. In the present sample, the names for the factors were informed by Jia et al. [14]. However, these are considered limited with regards to their wording for the dimensions of sleep with respect to the official PSQI metrics and those commonly used in research related to sleep health and clinical practice [1, 16, 35]. For example, it does not account for the regularity of sleep or napping. Nor does it differentiate between work- and free-days, so components associated with chronotype and social jetlag are not represented. Furthermore, the magnitude of specific external social stressors is not captured by the PSQI. Previous research has highlighted changes to such dimensions of sleep are important and were found to change within lockdown situations [4, 17]. In the future it will be important to determine more suitable concepts which reflect sleep under different circumstances and living conditions. Such approaches may also create new opportunities to diagnose sleep problems and improve aspects of sleep quality in a more individually tailored way. A final consideration is that the PSQI as well as other subjective sleep measures do not always reflect objective sleep measures such measures of sleep latency and fragmentation as recorded and defined using polysomnography or actigraphy [36]. This highlights the importance of using mixed methods and multiple measures for assessing sleep status. An assessment of the factor validity of the PSQI in research alongside objectives measures may be of interest.

## Conclusion

This study is the first to examine the PSQI structure validity during the unique context of the COVID-19 lockdown in a NZ working female cohort. Overall, the findings suggest the PSQI two-factor model was statistically superior to the original one-factor model and a three-factor model in detecting sleep impairment among working NZ females within a pandemic context. Although the factor structure requires further validation in other crisis situations across different populations, it indicates that internal validity of subjective sleep quality is better assessed by a PSQI two-factor model in line with some previous findings. This highlights the need to consider the different contexts in which the PSQI is used and its factor structure to differentiate between ‘good’ and ‘poor’ sleepers, as well as consider the weighting and interpretation of the PSQI global score.
